# Musculoskeletal ultrasound (MSK-US) in pediatric rheumatology: European preliminary results of the survey of the Pediatric Ultrasound Group of the Omeract Ultrasound Task Force

**DOI:** 10.1186/1546-0096-9-S1-P47

**Published:** 2011-09-14

**Authors:** Silvia Magni-Manzoni, Johannes Roth, Paz Collado, Esperanza Naredo, Maria Antonietta D'Agostino, Pietro Merli, Valentina Muratore, Sandrine Jousse-Joulin

**Affiliations:** 1 Fondazione IRCCS Policlinico S.Matteo, Pavia, Italy; 2 Children's Hospital of Eastern Ontario, Ottawa, Canada; 3 Department of Rheumatology, Hospital Severo Ochoa, Madrid, Spain; 4 DpRheumatology,Université deVersailles St-Quentin-en Yvelines, Ambroise Paré Hospital, Boulogne-Billancourt, France; 5 DpPediatrics, Fondazione IRCCS Policlinico S.Matteo, Pavia, Italy; 6 Rheumatology Unit and Immunology Laboratory, Université de Bretagne Occidentale, Brest, France

## Background

Despite the growing interest and use of MSK-US in children, its current use in paediatric rheumatology is not known.

## Objectives

To identify the current use of MSK-US and the areas most suitable for its development and standardisation in paediatric rheumatology.

## Methods

A questionnaire of 10 single or composite questions, including professional data, current use in daily practice, current clinical relevance of the main features of MSK-US, and areas for prospective development, has been sent to the members of PRINTO/PRES.

## Results

92/389 (24%) answers have been collected from 37 countries. The responders are mainly pediatric rheumatologists (80%), have a long-lasting clinical experience in paediatric rheumatology (74% >10 years), and are more clinicians (>70%) than researchers (24%).

MSK-US is used in clinical practice by>90%: personally by 40%, 49% by the radiologist, 16% by the adult rheumatologist. The most relevant features of MSK-US are the high patient’s acceptability (76%), the immediate improving of diagnosis of joint and soft tissue disease (73%), the assessment of synovitis and tendons/tendons’sheaths (73% and 70%), and the support to imaging guided joint injections (67%). The anatomical sites best suited for MSK-US are hips (87%), ankles (78%), wrists (65%), knees (64%), and mid-foot (63%). MSK-US is considered important for diagnosis, therapy monitoring, and research (70%).

## Conclusions

We identified the current use of MSK-US in paediatric rheumatology among the European network of PRINTO/PRES. The results outline the major reasons and areas of interest, useful for future steps towards a wider international standardized development of MSK-US in paediatric rheumatology.

